# The influence of minority stress-related experiences on mental wellbeing for trans/gender-diverse and cisgender youth: a comparative longitudinal analysis

**DOI:** 10.1098/rsos.221230

**Published:** 2023-07-26

**Authors:** Louise Black, Neil Humphrey, Jose Marquez

**Affiliations:** Manchester Institute of Education, University of Manchester, Manchester M13 9PL, UK

**Keywords:** adolescence, wellbeing, minority stress theory, gender identity

## Abstract

Trans and gender-diverse (TGD) adolescents are likely to experience poorer mental health and wellbeing than their cisgender peers. Minority stress theory has developed as a possible explanation for some of this disadvantage: factors such as increased bullying and discrimination lead to excess stress and reduced wellbeing. However, the evidence base remains limited. This study drew on secondary data analysis of the #BeeWell longitudinal cohort over 2 years (*N* = 26 042, aged 12–13 at time one, T1). We report two unregistered hypotheses relating to T1 (autumn 2021) data which was available at the time of stage-one submission: H1, mean differences in T1 wellbeing; H2, mean differences in T1 minority-related stressors. These are followed by two registered hypotheses relating to T2 (autumn 2022) data: H3, replication of T1 mean differences in T2 wellbeing; H4, predictions were made about the strength of the association between T1 minority-related stressors, controlling for sexuality and T2 wellbeing across T1 gender identity groups. At both time points cis-females, TGD and those who preferred not to say their gender had lower wellbeing than cis-males (CM), with the largest effect evident for the TGD group. TGD adolescents also showed the largest disadvantage (mean difference) compared with CM for minority stressors. Counter to H4 and minority stress theory, gender was not found to moderate the effect of minority stressors on later wellbeing. Our findings highlight the vulnerability of the TGD group in terms of wellbeing and minority stressors and are discussed with relevance for policy and future research.

## 1. Introduction

Inequalities are systematic, avoidable and unfair differences in outcomes between different populations or groups [1]. This paper focuses on inequalities, or the disadvantage in mental wellbeing among trans and gender-diverse (TGD) adolescents. ‘Trans’ refers to those who's gender identity is not aligned with their biological sex, and ‘gender-diverse’ refers to those who identify in another way than the traditional gender binary. Drawing on a contemporary longitudinal dataset (#BeeWell), we make a novel contribution to knowledge by assessing whether minority stress-related experiences (e.g. experiences of bullying and discrimination, lack of parent/carer support and feeling unsafe in one's local environment) influence later adolescent mental wellbeing, and the extent to which this varies by gender identity in ways consistent with the minority stress model [2]. We present the results of analyses for which data were already available prior to registration (pertaining to gender-related inequalities in mental wellbeing and minority-related stressors at the first timepoint of our study, T1) and registered confirmatory hypotheses that drew on data yet to be accessed before stage-one submission (pertaining to gender-related disparities in mental wellbeing in the second timepoint (T2), and the relationship between exposure to T1 minority-related stressors and T2 mental wellbeing).

What constitutes mental health and wellbeing in adolescence is poorly defined [3]. In a recent systematic meta-review, Black *et al.* [3] found that many indicators (including happiness, worry and loneliness) were found across subdomains (e.g. symptoms, positive wellbeing and quality of life), and in most measures. Furthermore, psychometric evidence was typically lacking, or failed to meet basic standards such as assessment of the structural properties needed for scoring, or understanding group differences [3–5]. Consideration of adolescent mental health and wellbeing must therefore be handled carefully in terms of selecting outcome measures and modelling structure. Based on these issues, and in-depth psychometric analysis of all mental health and wellbeing measures in the #BeeWell dataset [6], we opted to focus on mental wellbeing via the Short Warwick Edinburgh Mental Wellbeing Scale. While there is often interest in disease burden from a policy perspective [7], this measure includes experiences relevant to mental disorder such as relaxedness [8], has favourable scoring properties and demonstrates more acceptable sex/age measurement invariance, particularly compared with the available symptom measure [6]. In addition, wellbeing has considerable utility in population mental health research [9], given that most young people would not meet diagnostic criteria for disorder [8,10], and dimensional symptom measures tend to show substantial floor effects in non-clinical populations, limiting their reliability and validity [11]. Wellbeing also predicts a range of salient outcomes, including health-related lifestyles, mortality and physical health [9]. Finally, it is strongly statistically related to mental ill health, particularly internalizing symptoms [10,12–14]. A focus on wellbeing may therefore offer broader insight [10] and has been highlighted as a particular gap to be addressed in relation to TGD and minority stress in a recent systematic review [15].

### 1.1. Adolescence as a developmentally significant period

Beginning around age 10 and ending around age 24, adolescence is marked by significant changes across multiple aspects of development [16]. It is also a sensitive period for mental health and wellbeing. Subjective wellbeing begins to decline at the end of childhood [17], and this trend continues into at least mid-adolescence [18]. Alongside this, there are notable increases in the prevalence of probable mental health disorders from childhood to adolescence [14]. Furthermore, the peak age-of-onset of lifetime cases of mental ill health is 14.5 years [19].

Early adolescence, ending around age 14, is also an important period in terms of gender identity development [20], while also marking the emergence, triggered by puberty, of sexual desire and arousal [21]. The transitional years leading up to mid-adolescence, ages 12–14, are the particular focus of our study, and there is evidence that TGD young people may be at particular risk of worse mental health and wellbeing in this sensitive period [22]. Improving our understanding of adolescent development and determinants of mental wellbeing in relation to gender identity is therefore a key research and public health priority [23–25].

### 1.2. Mental health and wellbeing inequalities among trans and gender-diverse adolescents

There is mounting evidence that TGD youth experience worse mental health and wellbeing outcomes than their cisgender peers [22,24,25]. For example, two recent systematic reviews by Connolly *et al*. [26] and Mezzalira *et al*. [27] revealed significantly higher rates of problems including depression, suicidality, self-harm and eating disorders among TGD compared with cisgender youth. Furthermore, while there is substantial interest in the mental health of LGBTQ+ adolescents as a whole community [27,28], several studies suggest TGD young people face greater problems even compared with their cis-LGB counterparts [24,29–32]. There is therefore a particular need to focus on more granular gender identity data: TGD adolescents appear to show the greatest risk, perhaps due to gender dysphoria over and above, for example, discrimination faced by the broader LGBTQ+ community (see also minority stress theory described below) [24,25].

Despite these findings and calls, research to date has often amalgamated gender and sexual minorities into a single ‘LGBTQ+’ group in analyses (e.g. [33]). Such approaches probably substantially limit current understanding, and there is therefore a clear need to collect and analyse more granular gender and sexuality data [34,35]. This issue is discussed below in more detail (see ‘Gaps in the existing literature’), with particular reference to minority stress studies. Furthermore, while some research has considered more nuanced approaches to gender and sexuality individually, there appears to be more large-scale work focusing on sexuality (e.g. [36,37]), rather than the much-needed studies of gender.

### 1.3. The influence of minority stress-related experiences on mental wellbeing of trans and gender-diverse adolescents

The minority stress model [2] is a theoretical framework that has been developed to explain how and why gender and sexual minorities (including TGD youth) experience worse mental health and wellbeing outcomes than their cisgender, heterosexual peers [38]. It predicts that experiences of prejudice, discrimination, identity concealment, expectations of rejection and internalized stigma among gender and sexual minorities result in excess stress, ultimately leading to reduced wellbeing [39–41].

Recent work has extended minority stress theory to consider more specifically the experiences of TGD youth as a minority group. This includes the proposition that the exclusionary effects of stressors associated with cisnormativity (and aligned practices, such as *misgendering*) are distinct from those associated with heteronormativity, and potentially more powerful [15,42,43]. Indeed, Tankersley *et al.* note that to young people, ‘being a sexual minority feels less stigmatizing than being a gender minority’ [15, p. 184]. These authors' systematic review [15] identified a number of risk factors for TGD youth mental health that connect directly to the stressors available in the #BeeWell dataset and are therefore examined in the current study, including bullying/victimization and discrimination.

### 1.4. Gaps in the existing literature

Despite some progress, the evidence base remains limited in several respects. First, research focusing specifically on the influence of minority stressors on mental health and wellbeing outcomes among TGD youth remains relatively scarce. Second, the evidence base to date focuses on mental health difficulties, with studies tending to consider mental disorder, rather than robust measures of mental wellbeing [15]. Third, previous work has been almost exclusively cross-sectional in nature, or had insufficient time between stress exposure and outcome, and has tended to fail to directly compare outcomes for cisgender adolescents [15]. The use of cross-sectional designs severely limits opportunities for causal inference [44] and is particularly vulnerable to confounders in the mediation analyses that are typically used [45].

Fourth, the existing evidence base remains rather narrowly focused, with studies frequently focusing on a single stressor (e.g. [46,47]). Accordingly, there is a clear need for research which incorporates a more wide-ranging assessment of the types of experiences that may act as mechanisms underpinning wellbeing inequalities for TGD youth.

Fifth, studies focusing on gender rarely control for sexual orientation (and vice versa; see for example [32,36]), limiting precision. Furthermore, approaches to grouping typically follow a ‘majority versus minority’ model (e.g. LGBTQ and cis-heterosexual). This is particularly problematic in relation to gender, since it ignores the fact that there are mental health and wellbeing inequalities between male and female adolescents (with females routinely reporting poorer outcomes than males) [9,14,48]. There are parallel disparities in relation to exposure to certain stressors, including for example higher reported experiences of gender-based discrimination [49] and lower perceptions of neighbourhood safety [50] among females. Accordingly, a more nuanced approach is required in which wellbeing inequalities and their underpinning mechanisms for TGD youth can be isolated from those based on sexual orientation, while recognizing the inherent disparities in exposure to stressors and wellbeing outcomes (and the relationship between these) that may exist even between majority groups.

In summary, despite growing concerns and evidence that TGD adolescents may experience poorer mental health and wellbeing, and theory to explain this, our current understanding is limited by an evidence base underpinned by relatively limited designs.

### 1.5. The current study

Given these issues and the initial evidence reviewed above, we set out to examine gender-related differences in mental wellbeing and minority-related stressors among young people over time. We also examined the relationship between exposure to minority-related stressors and later wellbeing while controlling for T1 sexuality. We address limitations in previous research in several ways. First, we draw on granular data and focus on cis versus TGD groups following best-practice guidelines [28], rather than, for instance, collapsing gender and sexuality minorities into a single group. To support parsimony, allow sufficient time to elapse for the proposed stressors to impact wellbeing among TGD youth, and for temporal precedence, we base gender groupings on T1 data only. Given well-established differences between girls and boys for the variables under study, particularly mental health and wellbeing [9,48], we also opted to analyse cis-males (CM) and cis-females (CF) individually, consistent with work focusing on disorder [51]. Second, we examine and accommodate measurement issues in wellbeing and minority stress-related variables (where possible), given that adolescent mental health and wellbeing data are known be challenging, and these issues are generally underexplored [3,52]. Third, we use a more robust modelling framework, including longitudinal multi-group analysis, rather than the cross-sectional mediation models others have employed, which can be more vulnerable to problematic inferences. This structural equation modelling (SEM) approach can explicitly handle measurement error, limiting bias in slope estimates compared with ordinary least-squares regression [53]. This approach also enables controlling for sexuality, thus acknowledging the importance of this for wellbeing and the minority stress model.

We analyse a subset of the #BeeWell study dataset, which we describe in more detail in the following section (see Method). This paper focuses on the longitudinal cohort, which comprises over 20 000 young people in Greater Manchester, England. This subset of the main sample is being tracked, with annual data points, from Year 8 (aged 12–13) to Year 10 (aged 14–15). At the time of our initial stage-one submission, the first wave of data had been collected (T1, collected in autumn 2021, when the cohort were aged 12–13), but the second wave (T2, to be collected in autumn 2022, when the cohort are aged 13–14), on which our two confirmatory hypotheses hinge, had not.

Given that some data were already collected prior to our pre-registration, the current study is split into T1 hypotheses (the results of which are reported in the stage-one submission) and registered hypotheses that draw on T2 (added at stage two). To keep the difference between registered and secondary analyses clearly distinct, and for transparency, we analysed and report the hypotheses that relate to T1 only prior to stage-one submission. The following unregistered T1 predictions were made:

— H1: CM will report significantly higher T1 wellbeing than CF and TGD;
— H2^1^: (a) TGD adolescents will show a bigger disadvantage in relation to CM (i.e. negative mean difference) in T1 gender discrimination than CF; (b) TGD adolescents will show a greater disadvantage compared with CM for bullying at T1 than those who are CF; (c) TGD adolescents will show a greater disadvantage compared with CM for T1 family support than those who are CF and (d) CM will report lower levels of feeling unsafe in their local area than TGD and CF groups.

Drawing on forthcoming T2 data we also registered the following confirmatory hypotheses (see also table 1):

— H3: The pattern of mean differences for T1 wellbeing will replicate at T2 (comparing each of the TGD and CF groups with CM);
— H4: Controlling for T1 sexuality, (a) CM will show a weaker relationship between gender discrimination and wellbeing than all other groups; (b) TGD adolescents will show a stronger relationship between bullying and wellbeing than those who are CM and CF; (c) TGD adolescents will show a stronger relationship between a lack of family support and wellbeing than those who are CM and CF; (d) CM will show a weaker relationship between feeling unsafe and wellbeing than all other groups.

**Table 1.** Registered hypotheses, methods, possible results and interpretations.

hypothesis	analysis	possible results	interpretations
Hypothesis 3: The pattern of mean differences found in H1 will replicate at T2 (comparing each of the CF and TGD groups with CM).	Assess whether latent means are significantly different and lower for groups other than CM accounting for any measurement non-invariance.	The pattern of mean difference replicates in terms of significance (fully or partially).	This would lend (full or partial) support to the pattern found at T1 and suggest stability across age groups studied.
		The pattern of mean differences does not replicate in terms of significance.	Suggests mean differences across gender groups may vary with age, which should be explicitly considered in future research. Alternatively, this may reflect problems with measurement, given known issues in this field.
Hypothesis 4: We predict MS variables to be more/less strongly predictive of wellbeing as follows: (a) CM < CF, TGD (gender discrimination) (b) TGD > CM, CF (bullying) (c) TGD > CM, CF (lack of family support) (d) CM < CF, TGD (unsafe)	Multi-group SEM—(i) evaluate whether structural parameters are substantially different (see description of MNCI threshold below); (ii) qualitative comparison of whether structural paths are stronger/weaker according to patterns a–d taking into account CIs.	Differences in structural paths are found across groups fully/partially consistent with patterns a–d.	MS variables play a more important role in adolescent wellbeing for certain groups than others.
		No differences in structural paths are found across groups.	MS variables play a similar role in predicting adolescent wellbeing across groups (though levels of these variables may vary (H1/H2).

*Note.* H1 = hypothesis 1; H2 = hypothesis 2; T2 = time 2; MNCI = McDonald's non-centrality index; CIs = confidence intervals, CM = cis-male; CF = cis-female; TGD = trans and gender-diverse (including non-binary); MS = minority stress.

### 1.6. Registered reports and secondary data analysis

While registered reports are typically used when no data has been collected, applications such as ours are valuable to capitalize on large longitudinal studies, when transparent and robust protocols are followed [54–57]. Specifically, for the present study, the following factors make a registered report appropriate: first, our registered hypotheses are confirmatory. Second, we proposed a relatively complex analysis pipeline to accommodate known measurement issues in this field [3,6]. This represents potentially increased researcher degrees of freedom, particularly since alternative modelling frameworks (e.g. mediation) could be used to address our hypotheses. The transparency afforded by the registered report format is therefore beneficial to progress in understanding the wellbeing of TGD adolescents and its antecedents.

Registered reports with secondary data can also pose particular risks in terms of researcher bias, which need to be addressed [55]. Prior knowledge of data can lead to bias because expectations are formed that lead researchers to pursue particular analyses after testing related questions with the same variables. We therefore declare our prior access and other analyses of the T1 #BeeWell dataset [6,22,58,59]. We note in particular our existing analysis that suggests disparities in mental wellbeing between gender and sexual minority adolescents and their peers (which serves as a key motivation for the current study; [22]), but also highlight that this pertains specifically to data gathered at T1 only, concerns a range of mental health and wellbeing outcomes and different approach to analysing gender, without any analysis of minority stressors that might underpin such inequalities.

## 2. Method

### 2.1. Design

#BeeWell uses a hybrid population cohort study design, comprising: (i) a truncated longitudinal study in which participants are tracked with annual data points from age 12 to 15 (e.g. from Year 8 to Year 9 to Year 10 of secondary school; Sample 1); and (ii) a cross-sectional study comprising annual data points for participants aged 14–15 (e.g. those in Year 10 of secondary school at a given data point; Sample 2). Our secondary analysis draws on the first (T1) and second (T2) annual data points for Sample 1. As mentioned, T1 data have already been collected and accessed by the authors for other work. T2 data were collected during a fixed window between 20 September and 2 December 2022, after the initial Stage-one submission of the paper. Reviews of this submission were received on 16 January 2023. However, the lead analyst's access to the T2 data was prohibited until in-principle acceptance of the revised manuscript.

### 2.2. Participants

The final sample after removing eight participants who had missing on all variables in the current study based on both time points was *N* = 26 042. The demographic characteristics of the study sample at both time points are summarized in table 2, alongside national averages, where available. The composition of the study sample and schools mirrors the population of young people in England with respect to sex, special educational needs (SEN), and whether their first language is known or believed to be English. However, there are differences in ethnicity (higher proportion of White pupils nationally) that probably reflect migration trends in several areas of the city-region over the last several decades [22].

**Table 2.** Sample composition and equivalent national data.

variable	frequency	national average
sex	F = 49.54%, M = 50.16%, missing = 0.3%	50.26% male, 49.74% female
gender T1	girl (including trans girl) = 30.45%, boy (including trans boy) = 31.32%, non-binary = 1.88%, I describe myself in another way = 2.43%, prefer not to say = 5.00%, missing = 28.92%	N/A
gender T2	girl (including trans girl) = 28.28%, boy (including trans boy) = 29.59%, non-binary = 1.06%, I describe myself in another way = 1.63%, prefer not to say = 3.06%, missing = 36.38%	N/A
sexuality T1	bi/pansexual = 5.62%, gay/lesbian = 1.94%, heterosexual/straight = 50.44%, I describe myself in another way = 3.47%, prefer not to say = 8.3%, missing = 30.23%	N/A
sexuality T2	bi/pansexual = 4.17%, gay/lesbian = 1.57%, heterosexual/straight = 49.62%, I describe myself in another way = 2.35%, prefer not to say = 5.25%, missing = 37.05%	N/A
ethnicity	any other ethnic group = 2.33%, Asian = 16.25%, Black = 5.80%, Chinese = 0.89%, mixed = 5.98%, unclassified = 2.11%, White = 64.17%, missing = 2.46%	2.20% any other ethnic group. 12.00% Asian, 6.20% Black, 0.50% Chinese, 6.30% mixed, 2.0% unclassified, 70.80% White
SEN	not SEN = 82.39%, SEN = 15.46%, missing = 2.15%	85.9% no, 14.1% yes
FSM	not FSM = 69.87%, FSM = 27.36%, missing = 2.77%	N/A^a^
T1 survey completion	77.72%, missing = 22.28%	N/A
T2 survey completion	68.85%, missing = 31.15%	N/A

*Note*. National data derived from Explore Education Statistics online tool [60].

^a^National data are not available for free school meal (FSM) eligibility in the last 6 years (known as EverFSM6). National data for current free school meal eligibility indicate that 20.9% of pupils aged 11–16 are eligible. The final two rows reflect the percentage of Year 8 at T1 and Year 9 at T2 considering all participants over both time points.

### 2.3. Measures

#### 2.3.1. Gender identity

The gender identity item was developed in consultation with #BeeWell Young Peer Reviewers and national LGBTQ+ organizations. To probe gender identity, participants were asked if they were a girl (including trans girl); boy (including trans boy); non-binary; describe themselves in another way; or, prefer not to say. Transgender status (e.g. transgender, cisgender) was noted where self-reported gender identity and linked administrative data on sex did not correspond.

Following best-practice guidelines to compare cisgender with aggregated trans groups [28], we opted to cross-reference adminstrative sex data with self-reported gender data to derive the following groups: CM, CF, TGD and prefer not to say (PNS; for frequencies table 3, which also provides frequencies of each of the binary sexuality groups used as a covariate). Though we did not make hypotheses about the PNS group, it was important to model this separately for several reasons: first, in the T1 dataset the frequency of missingness for gender was equivalent to other variables (see below), suggesting the PNS response is different to other missingness and should be handled separately; second, studies suggest PNS is associated with privacy concern [61–63], suggesting analyses could be biased by combining them with another group; third, this privacy concern may mean this category could provide tentative insight into those who are questioning (i.e. the Q in LGBTQ+) or not yet out, though much more work to understand the relationship between being out and survey responses in young people is needed.

**Table 3.** Overview of time one gender and sexuality groups^2^.

group	*N*
cis-males (CM)	7914
cis-females (CF)	7732
trans and gender-diverse (TGD)	1549
prefer not to say (PNS)	1300
missing on gender	7547
heterosexual	13 136
sexual minority	2872
missing (including prefer not to say) on sexuality	10 034

How groups were defined can be seen in detail in the accompanying R code.

#### 2.3.2. Mental wellbeing and minority stressors

Table 4 provides information on the measures used in our analyses. For H1, H3 and H4 wellbeing is the outcome, and for H4 all other variables described in table 4 are used as predictors, considering sexuality as a covariate. Sexuality was also recoded to heterosexual versus sexual minority to avoid adding many dummy variable paths at a substantial power cost.

**Table 4.** Overview of wellbeing and minority stressor measures.

construct	measure	no. items	sample item	response format	latent or observed
mental wellbeing	Short Warwick Edinburgh Mental Wellbeing Scale (SWEMWBS) [64]	7	‘I've been feeling useful’	none of the time, rarely, some of the time, often, all of the time	latent
bullying victimization	adapted from understanding society and health behaviours in schools checklist [65,66]	3	‘How often do you get physically bullied at school? By this we mean getting hit, pushed around, threatened, or having belongings stolen’	not bullied at all, not much (one–three times in the last six months), quite a lot (more than four times in the last six months), a lot (a few times every week)	latent (structural model only)
gender discrimination	item adapted from determinants of adolescent social wellbeing and health study & Harvard measuring discrimination resource [66,67]	1	‘How often do people make you feel bad because of your gender?’	often or always, some of the time, occasionally, hardly ever, never	observed
lack of parent/carer support	student resilience survey [68]	4	‘At home there is an adult who listens to me when I have something to say’	1 = never to 5 = always	latent
feeling unsafe in local area	adapted from health behaviours in schools checklist [65,66]	1	‘I feel safe in the area where I live’	strongly agree, agree, neither agree nor disagree, disagree, strongly disagree	observed
sexuality	developed in consultation with #BeeWell Young Peer Reviewers and national LGBTQ+ organization	1	'What best describes you?'	bi/pansexual; gay/lesbian; heterosexual/straight; describe myself in another way; or, prefer not to say	observed (collapsed to binary for parsimony)

### 2.4. Statistical analysis

Other than for observed mean comparisons, a SEM framework was used, underpinned by the following estimation and fit decisions.

#### 2.4.1. Estimation

The robust maximum-likelihood (MLR) estimator was used so that missing data could be handled via full information, given the outcome and most survey variables are measured on a five-point response format [69], and estimators designed for categorical data have tended not to be used or recommended when setting thresholds for approximate invariance testing [70,71] (which was preferred given the problems with large sample sizes and exact difference testing [72]).

#### 2.4.2. Model fit

Consistent model fit cut-offs are not applicable across all modelling scenarios since these are sensitive to issues such as sample size and model type. However, methods to simulate bespoke cut-offs are not readily available for non-normal data, or structural models [73]. Therefore, for simplicity, we judged models against conservative often-used cut-offs, confirmatory fit index (CFI) > 0.95, root mean square error of approximation (RMSEA) < 0.06, standardized root mean square residual (SRMR) < 0.08 [74].

#### 2.4.3. Pre-analysis checks

Data quality checks and cleaning of T2 data were conducted by the #BeeWell data manager (not one of the authors of this study) following the procedure also used for T1. This involved removal of duplicate cases, removal of cases with so few responses no scores could be computed (across all variables, not just those in the current study), removal of responses below a minimum time and removal of cases with the same response across all items.

Since non-normality was accommodated via MLR in the SEM analyses, and most data were analysed at the item level (via latent variables) and are therefore also on a short ordinal scale (maximum 1–5), we did not search for or remove statistical outliers. The distributions of T2 wellbeing item categories were checked to ensure they were similar to T1, and less than 50% ceiling effects were observed to ensure extreme asymmetry was avoided, which would make MLR estimation unsuitable [69]. It was anticipated distributions would be similar at T2 but should the aforementioned conditions not be met, the weighted least squares with means and variances adjusted would need to be employed instead.

### 2.5. Time one hypotheses

#### 2.5.1. Hypothesis one

***Measurement invariance.*** In order to make valid mean comparisons, measurement invariance of the wellbeing outcome across gender groups was analysed. Given that there was a large discrepancy between group sizes, and this can affect the accuracy and power of invariance testing, we adopted the resampling approach laid out by Yoon & Lai [75], in which 100 datasets are resampled and analysed with each group rebalanced to the smallest group size. We proposed a comprehensive pipeline to assess measurement invariance but as soon as results indicated a model could be taken forward, further testing halted. The first step was to estimate a baseline model in each of the groups separately. If acceptable fit was not achieved in any of the groups individually, modification indexes (for residual covariances, given local independence can be unrealistic for mental health; [76]) were examined to see if acceptable fit could be achieved. Following acceptable fit (based on cut-offs listed above), for baseline models, a configural model (free loadings and intercepts) followed by a scalar model (constrained loadings and intercepts) was estimated. For simplicity, metric invariance (constrained loadings only) was not analysed: this stage is considered to be the most unrealistic criterion [77], the proposed subsequent alignment analysis provides insight into the invariance of both loadings and intercepts, and using alignment in this way rather than successive testing avoids capitalizing on chance via modification indices as is typically then conducted to find a partially invariant solution. The scalar model was then compared with the configural model via McDonald's non-centrality index (MNCI), with a cut-off of ΔMNCI −0.01, since this has been shown to perform well for measurement and structural invariance testing (allowing us to employ a consistent approach across analyses, see also below; [70]). Using this approximate fit index comparison can reduce false positive differences between groups. We also adopt the more lenient criterion suggested by Kang *et al*. [70] across our analyses given our provision for alignment testing, and to avoid over-strictness in the structural model which has many paths and groups.

If the difference between the scalar and configural model exceeded ΔMNCI −0.01, alignment analysis was conducted using free optimization in the first instance, given that more than two groups were compared, followed by fixed if indicated by a warning [78]. If any loading or intercept parameters remained significantly non-invariant, even after alignment optimization, these were freed in a partially invariant model. The fit of the partially invariant model was then compared again with the configural model to ensure the ΔMNCI −0.01 cut-off was satisfied.

***Latent mean comparison.*** If full scalar invariance was achieved, this model could be used to assess whether CM have significantly higher mean levels of wellbeing. In the scalar model the mean of the first group, specified to be CM, was fixed at 0 while the others were freely estimated, providing a *p*-value and effect size for the difference to CM. If scalar invariance was not supported, but the aligned model showed no significant additional non-invariance, the same procedure described for the scalar model was used. Similarly, if a partially invariant model was required, this could be used in the same way to consider whether there were significant differences between latent means. In each of these scenarios, the resulting estimate for each group other than CM can be interpreted as Glass's Δ, a member of the *d* family of effect sizes [79]. Given that multiple mean comparisons were conducted (each group against CM), these were adjusted using the false discovery rate (also known as the Benjamini–Hochberg procedure; [80]).

#### 2.5.2. Hypothesis two

***Latent minority stress variables.*** For (lack of) family support, the same procedure as for H1 was used. First this involved measurement invariance testing with the following steps as appropriate based on the result of each stage: baseline models, modification indices, configural model, scalar model, alignment analysis and partial model (see also above). Second, the most appropriate of these models was used for mean comparison as described above.

***Observed minority stress variables.*** For the observed minority stress variables, gender discrimination, feeling unsafe and bullying^3^, unpaired Welch's *t*-tests comparing each group with CM were conducted [81]. Variables were coded to represent more stress, e.g. feeling *un*safe.

### 2.6. Registered time two hypotheses

**Hypothesis three.** The same procedure outlined for H1 was used: measurement invariance (using resampled balanced groups) was explored in order to accommodate any non-invariance for valid mean comparison. The following steps were taken as appropriate: baseline models, modification indices, configural model, scalar model, alignment analysis and partial model. Then, the most appropriate of these models was used for mean comparison, again comparing each group with CM.

**Hypothesis four.** The full measurement model, informed by group invariance analyses described above and longitudinal invariance of the outcome, was assessed prior to the final longitudinal SEM, in order to confirm conclusions about change in the outcome could be robustly drawn [82].

***Wellbeing longitudinal invariance.*** A similar approach to the multi-group measurement invariance analyses described above was followed for longitudinal invariance. First, baseline models in each time point separately were estimated. If these met acceptable fit, including additional free residual covariances based on modification indices where necessary, a configural model (free loadings and intercepts) was estimated. Since Mplus alignment analysis cannot be implemented with longitudinal data [78], the metric invariance stage (constrained loadings only) was included. If this met the ΔMNCI −0.01 cut-off, the scalar model was estimated and then compared with the metric model. Where necessary to meet the ΔMNCI −0.01 criterion, parameter constraints were freed across time points based on modification indices (both metric and scalar stages). If partial group invariance was needed for each of T1 and T2 wellbeing, longitudinal invariance was analysed incorporating freed parameters (across particular groups) into the longitudinal model.

***Full measurement model.*** Assuming longitudinal measurement invariance was supported, or could be accommodated via partial invariance, these constraints were incorporated into a full measurement model of all latent constructs (T1 and T2) in which constraints across gender groups were also imposed (via multi-group confirmatory factor analysis), except for any non-invariant parameters identified in H1 and H3. The fit of this model was considered before proceeding to the structural model.

**Figure 1.**
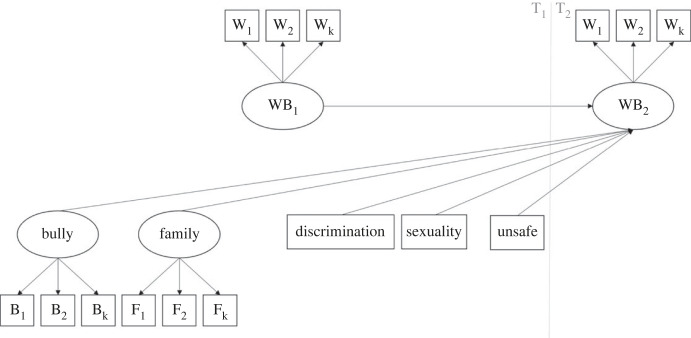
Conceptual diagram of structural model (hypothesis 4). Note: WB = wellbeing; W = wellbeing item; B = bullying item; F = family item. This was estimated in each group in a multi-group model as described. For ease of interpretation, some paths, e.g. number of items and predictor covariances, have been omitted.

***Multi-group SEM.*** The T1 latent and observed predictors analysed in H2 and T1 wellbeing were added to the multi-group measurement model, such that each predicted T2 wellbeing (figure 1). These structural parameters were first allowed to vary across groups, and then constrained to be equal. If the ΔMNCI −0.01 cut-off was exceeded between these two models, structural paths in the free model were qualitatively considered to see whether predictions in H4 are upheld. We planned to draw on 95% confidence intervals and only interpreted differences between pairs of paths if these did not overlap. If the ΔMNCI −0.01 cut-off was not exceeded, the null hypothesis that there are no differences for each of the structural paths of H4 was accepted.

### 2.7. Time two power

We present an *a priori*, simulation-based power analysis in order to delineate, in the absence of clear theoretical or practical boundaries on meaningful effect sizes, the smallest effect size that we have sufficient power to detect, focusing on the structural paths in H4, the most complex model [83]. Setting such a minimal effect size is also important due to increased type I error rates in underpowered studies as well type II [84].

Population parameters were informed by average parameters in available T1 data (wellbeing and minority stress variables, see electronic supplementary material, R code), with latent correlations and the path between T1/T2 wellbeing set at 0.45, correlations involving observed variables at 0.30 and loadings at 0.70. Item residual variances were therefore set at 0.51. The simulation study was conducted in Mplus using the MLR estimator. Missingness was set at 10% for T1 variables based on preliminary analysis which showed this to be the average, and at 20% for T2 wellbeing, given that some attrition was expected. Simulations suggested that 80% power could be achieved for structural paths as small as 0.12 in the smallest group. We therefore consider 0.12 the minimal detectable effect size in the smaller groups (TGD, *N* = 1550, and PNS, *N* = 1282). In the larger groups (CM, *N* = 7895, and CF, *N* = 7716), 80% power was achieved for effects as small as 0.05. We include the output of the simulation demonstrating these effects in the electronic supplementary material.

## 3. Exploratory time one results

After removing cases with missing on all T1 wellbeing and minority stress variables, a sample of *N* = 20 242 was used in the H1 and H2 analysis. The grouping variable was missing for 1746 participants, further reducing the effective sample.

### 3.1. Time one wellbeing invariance and mean differences: hypothesis one

Baseline models met acceptable fit thresholds (see electronic supplementary material, table S1). Model fit for configural and scalar models can be seen in electronic supplementary material, table S2, with ΔMNCI showing support for scalar invariance (i.e. measurement parameters could be constrained across groups). Mean differences for T1 wellbeing derived from the scalar model can be seen in table 5.

**Table 5.** Latent mean differences for T1 wellbeing.

group	mean [95% confidence intervals]	*p*
CM	0	–
CF	−0.27 [−0.33, −0.20]	<0.001
TGD	−0.56 [−0.63, −0.49]	<0.001
PNS	−0.33 [−0.40, −0.26]	<0.001

*Note*. CM = cis-males; CF = cis-females; TGD = trans and gender-diverse; PNS = prefer not to say. The mean in CM is fixed to 0 to allow comparison. *p*-values are adjusted using the false discovery rate.

### 3.2. Time one minority stress mean differences

#### 3.2.1. Lack of family support

Baseline models met acceptable fit thresholds (see electronic supplementary material, table S1). Model fit for configural and scalar models can be seen in electronic supplementary material, table S2. Since the ΔMNCI threshold was met, mean comparison could therefore be drawn from the scalar model (table 6).

**Table 6.** Latent mean comparisons for family support.

group	mean [95% confidence intervals]	*p*
CM	0	–
CF	−0.04 [−0.11, 0.04]	0.38
TGD	−0.46 [−0.55, −0.36]	<0.001
PNS	−0.38 [−0.47, −0.29]	<0.001

*Note*. CM = cis-males; CF = cis-females; TGD = trans and gender-diverse; PNS = prefer not to say. The mean in CM is fixed to 0 to allow comparison. *p*-values are adjusted using the false discovery rate.

#### 3.2.2. Observed minority stress variables

The results of mean comparisons for observed minority stress variables can be seen in table 7.

**Table 7.** Results from Welch's *t*-tests and Cohen's *d* for observed minority stress variables compared with cis-males.

group	*t*	d.f.	mean other group	mean CM	Cohen's *d* [95% CI]
gender discrimination					
CF	18.16**	13 664.24	1.43	1.20	0.30 [0.27, 0.33]
TGD	27.44**	1561.36	2.29	1.20	1.28 [1.22, 1.34]
PNS	10.53**	1279.25	1.55	1.20	0.48 [0.41, 0.54]
feeling unsafe					
CF	1.35	14 787.42	1.91	1.89	0.02 [−0.01, 0.05]
TGD	11.62**	1960.77	2.24	1.89	0.36 [0.3, 0.42]
PNS	5.52**	1496.8	2.07	1.89	0.19 [0.13, 0.25]
bullying					
CF	5.04**	14 578.89	4.10	3.97	0.08 [0.05, 0.12]
TGD	15.08**	1767.64	4.90	3.97	0.53 [0.48, 0.59]
PNS	6.32**	1427.73	4.35	3.97	0.23 [0.16, 0.29]

*Note*. d.f. = degrees of freedom; CI = confidence interval; ** *p* < 0.001. *p*-values were adjusted using the false discovery rate. CM = cis-males; CF = cis-females; TGD = trans and gender-diverse; PNS = prefer not to say.

## 4. Results for registered analyses

Ceiling effects for wellbeing items were evaluated and were well below the 50% criterion set out in our stage-one submission (maximum = 14.72% for category five of the last SWEMWBS item), allowing us to proceed with MLR estimation (missingness ranged from 28.98 to 38.57%).

### 4.1. Hypothesis three: time two wellbeing differences

In terms of invariance testing drawing on rebalanced samples (prior to mean difference estimation), baseline models in each group separately resulted in acceptable fit (see electronic supplementary material, table S3) consistent with T1 results.

Model fit for configural and scalar models can be seen in electronic supplementary material, table S4, with ΔMNCI showing support for scalar measurement invariance across the four gender groups. Mean differences for T2 wellbeing derived from the scalar model can be seen in table 8.

**Table 8.** Mean differences across gender groups for time two wellbeing.

group	mean [95% confidence intervals]	*p*
CM	0	–
CF	−0.33 [−0.42, −0.25]	<0.001
TGD	−0.56 [−0.65, −0.46]	<0.001
PNS	−0.32 [−0.41, −0.23]	<0.001

*Note*. CM = cis-males; CF = cis-females; TGD = trans and gender diverse; PNS = prefer not to say.

The pattern of mean differences closely replicated the T1 wellbeing findings, with similar effects for each group, and the largest disadvantage between CM and TGD groups (−0.56 at both time points). At T1, the CF disadvantage (−0.27) was slightly smaller than that for PNS (−0.33), while at T2, the CF disadvantage (−0.33) was slightly greater than PNS (−0.32). However, at both time points, the confidence intervals for CF and PNS overlapped, and point estimates were equivalent to the first decimal place. We consider the pattern to be clearly supported for TGD versus CM, and CF and PNS to have similar disadvantages to one another at both time points.

### 4.2. Hypothesis four: longitudinal effects of minority stress variables across gender groups

As per our stage-one submission, we analysed longitudinal invariance of the outcome, which was upheld (while also constraining group invariance in line with H1 and H3 scalar models, see electronic supplementary material, table S5). We then proceeded to estimate the full measurement model, which showed acceptable fit according to our registered criteria for CFI, RMSEA and SRMR, $\chi_{818}^{2}=4354.52$, *p* < 0.001, CFI = 0.962, RMSEA = 0.031, MFI = 0.889, SRMR = 0.035. Contrary to H4, structural invariance held across all four groups for the multi-group longitudinal model (table 9). This means that gender was not a significant moderator on the effects of minority stress variables on wellbeing.

**Table 9.** Fit statistics for structural invariance for minority stress predicting wellbeing across gender groups.

model	*χ*^2^	d.f.	*p*	CFI	RMSEA	SRMR	MNCI	ΔMNCI
unconstrained	4936.02	1022	<0.001	0.960	0.029	0.032	0.878	–
constrained	4967.47	1040	<0.001	0.960	0.029	0.034	0.878	<0.001

The structural paths for minority stress variables predicting T2 wellbeing for the constrained multi-group model can be seen electronic supplementary material, table S6.

**Figure 2.**
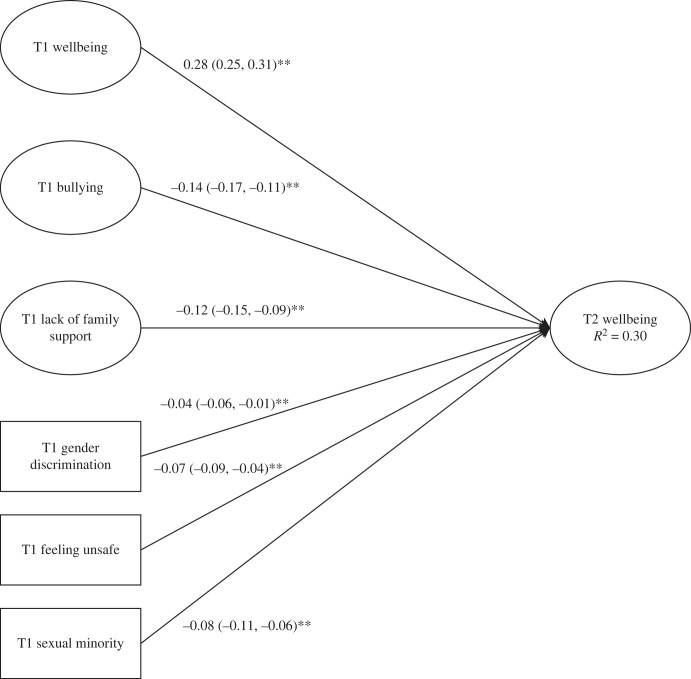
Structural paths for single-group model. Note: ellipses represent latent variables (those measured by several items), estimated accounting for measurement error, while rectangles represent single observed items containing measurement error.

Though we did not plan for this in our stage-one submission, we opted to re-estimate and additionally report the structural model treating the whole sample as a single group (but still maintaining longitudinal constraints), given the finding that measurement and structural and measurement parameters were invariant and to afford greater power. The finding of structural invariance itself provides support for this: the structural paths between minority stressors and wellbeing did not vary as a function of gender and there was therefore no need to treat gender groups separately and then constrain them to equality. The finding of structural invariance was not only surprising theoretically, but also, arguably, statistically since we employed a strict omnibus test (i.e. we looked for differences between all groups simultaneously), and we therefore planned to consider qualitative differences. We had therefore failed to plan for the eventuality of structural invariance in our stage-one registration. In the light of the models' similarity in terms of format, and the greater parsimony afforded by the single-group model, we focus on the latter in the main paper. Nevertheless, the multi-group model (implicitly consistent with our registration) is presented in electronic supplementary material, table S6, as mentioned above. In addition, the constrained multi-group paths were often below the minimum effect size determined by our *a priori* power analysis. Given measurement and structural invariance, we therefore opted to focus on interpreting the single-group model effects in our discussion. The single-group model showed acceptable fit, $\chi_{236}^{2}=3475.990$, *p* < 0.001, CFI = 0.972, RMSEA = 0.023, SRMR = 0.026, MFI = 0.922 with the variance explained in T2 wellbeing *R*^2^ = 30% (structural paths can be seen in figure 2).

## 5. Discussion

The current study drew on secondary data and reported two unregistered (H1 and H2, for which data were available at the time of stage-one submission), and two registered hypotheses (H3 and H4 for which data were only accessed after in-principle acceptance). We considered mean differences in wellbeing and minority stressors (bullying, lack of family support, gender discrimination and feeling unsafe in the local area), comparing CM with each of CF, TGD and prefer not to say (PNS) adolescents (H1–H3). We also examined the relationship between these variables across two time points and whether these varied by gender (H4). We drew on a large dataset with granular gender data, collected based on consultation with experts and young people, a robust measure of wellbeing, analysed to account for measurement error, and controlled for sexuality in our longitudinal analysis.

Consistent with our predictions about mean differences, TGD youth consistently showed the greatest wellbeing disadvantage (mean difference) compared with CM at both time points, and the pattern of wellbeing inequalities considering all groups was broadly consistent across time (H1 and H3). Moreover, the size of the difference in wellbeing between CM and TGD groups appeared substantial. In terms of minority stressors, the TGD group was also consistently subject to the largest inequalities when compared with the CM group, with a particularly marked disparity for gender discrimination (H2). In contrast with H1–H3, we did not find evidence to support the following from Meyer's seminal paper (H4): ‘Of course, minority identity is not only a source of stress but also an important effect modifier in the stress process' [2, p. 678]. On the basis of this tenet of minority stress theory, we had predicted that gender groups would show different relationships between minority stress variables and wellbeing over time. This hypothesis was *not* supported, with evidence of measurement and structural invariance across all four gender groups. We therefore did not reach the stage of our analysis plan in which we would compare paths across groups, since, surprisingly, the strict omnibus test for differences between any group was met.

In summary, wellbeing and minority stressors showed hypothesized and sometimes substantial group differences across gender groups, with TGD adolescents particularly disadvantaged. However, while all four minority stress variables predicted later wellbeing, the magnitude of these relationships was not moderated by gender.

### 5.1. Differences in wellbeing

We begin by discussing the stark differences in wellbeing identified between CM and TGD groups. Contextualizing the size of this effect is somewhat challenging, given the scarcity of studies considering wellbeing by gender (beyond the traditional binary) in general population samples. Nonetheless, Clark *et al.*'s [85] research provides a useful frame of reference given numerous methodological parallels to the current study (focused on young people; consideration of gender beyond male/female and use of a large, representative sample). These authors noted larger disparities in wellbeing than those reported here (their odds-ratio of 5.7x for TGD youth versus cisgender youth equates to *d* = 0.96 [86]), though this difference could be amplified due to measurement and analytic considerations (e.g. use of clinically significant symptoms of depression as the outcome threshold and focus on transgender status). An alternative approach to contextualization, given our focus on minority stress theory, is to compare our TGD wellbeing disparities with those reported for sexual minority youth. Two meta-analytic reviews [87,88] reported standardized mean differences of 0.33–0.39 (for symptoms of depression). Comparison of our TGD finding with these aggregated studies is therefore consistent with literature that indicates TGD young people face greater problems even compared with their cis-LGB counterparts [24,29–32].

When comparing the other groups, CF and PNS, with CM, we found smaller wellbeing inequalities than were evident for TGD youth (*d* ≈ 0.32 for both groups at both timepoints). With reference to CF, this finding is similar to sex-based disparities in wellbeing reported by Patalay & Fitzsimons [13] (standardized mean difference ≈ 0.20 depending on outcome and considering 95% confidence intervals). However, a recent international analysis of over 500 000 adolescents from 73 countries, found binary, self-reported gender differences to vary substantially by general mental health/wellbeing outcome (standardized mean difference range of 0.04–0.46) [89].

The above issues suggest future work might therefore consider groupings as we did based on best practice [28] and use robust measures of mental health or wellbeing. Notably, brief symptom measures applicable in large-scale research can be particularly vulnerable to male/female non-invariance [6], and are generally poorly validated [3]. The current study therefore makes a major contribution in that it demonstrated theorized inequalities using a robust measure with established measurement invariance, thought to be more appropriate to general population samples [10]. In any case, the current study confirms the need for policy and practitioner attention to this group, as well as the research community [25]. In addition, considering the substantial body of literature that has focused on the lower wellbeing of girls compared with boys, future work should consider how robust widely cited male/female inequalities are under more appropriate wellbeing *and* gender measurement conditions.

In addition to the above, the evidence of PNS versus CM wellbeing inequalities is something that warrants focused attention in future research, especially given that these were similar to those observed for CF versus CM, and represented a non-negligible proportion of the sample. Future work might consider the extent to which this category is used as an option for those who are questioning their identity (i.e. the Q in LGBTQ+), those who already identify as TGD but are not yet comfortable with selecting that response, or whether a heterogeneous group select this due to elevated privacy concerns [61–63]. If insight into who selects this response can be gained, future research could also consider whether there is an additional disadvantage for minority youth who feel unable to disclose their identity.

### 5.2. Differences in minority stressors

Consistent with H2a-d, TGD adolescents were subject to the largest disadvantages (compared with CM) for all minority stress variables (exposure to bullying *d* = 0.53, lack of family support Δ = 0.46 and feeling unsafe in their local area *d* = 0.36, similar to findings by Clark *et al*. [85]). For H2b-c (bullying and lack of family support), we expected CF not to show particular disadvantage compared with CM. In line with these predictions, no significant differences between CF and CM were found for family support, though a statistically significant effect was found for bullying (CF reporting higher exposure to bullying than CM). However, the size of this effect (0.08) is arguably negligible, particularly since this is a comparison between the two largest groups. Finally, as expected, CF reported significantly greater gender discrimination than CM, though the magnitude of this effect (*d* = 0.30) pales in comparison with the equivalent effect for TGD youth (*d* = 1.28).

The fact that gender discrimination was by far the most prominent minority stressor, yielding the largest disparity between TGD and CM (and to a lesser extent PNS, followed by CF), is consistent with recent gender-focused extensions of minority stress theory [42,43]. Such work has highlighted the predominance of cisnormativity (the assumption that being cisgender is or should be the norm), the marginalizing social norms it reflects and exclusionary practices it produces (e.g. misgendering and non-affirmation in which gender is not supported or recognized) that create prejudicial conditions for TGD adolescents.

Gender discrimination should therefore be considered a salient problem in and of itself, one which probably particularly faces TGD adolescents, and to a lesser extent CF and PNS groups. Gender discrimination also significantly predicted later wellbeing, though to a lesser extent than multiple-indicator predictors (bullying and parent/carer support). Based on these findings, and taking into account likely downward bias for single indicator predictors [90], we recommend further multiple-indicator research for minority stress research considering gender discrimination and wellbeing in particular.

### 5.3. Implications for minority stress theory

As noted above, we found evidence of higher levels of minority stressors among TGD adolescents, and our registered analysis demonstrated that these stressors at T1 were predictive of poorer mental wellbeing at T2. However, counter to our hypothesis, *these affected later mental wellbeing in a uniform way, regardless of gender*. Based on this and relevant literature, we highlight several key issues for minority stress theory and adolescence.

Although Meyer's [2] original formulation of minority stress theory explicitly predicts that (minority) identity would moderate the effect of minority stressors on mental health, we are not aware of any previous studies that have conducted such moderation analysis. This is perhaps unsurprising given the issue identified in the Introduction that very few studies have properly considered gender, particularly comparatively with cisgender peers. In addition, the theory is not clearly linked to testable models, as is typical in psychology [91]. Nevertheless, this moderating effect is somewhat fleshed out, for instance in the speculation that expectations of victimization/rejection and increased vigilance may contribute to mental health over and above actual levels of the stressor [2,42]. Given the secondary nature of our analysis and to some extent power considerations, we only looked at narrow set of minority stress variables. Since we have demonstrated a robust framework for considering the moderation hypothesis of minority stress theory, which to our knowledge has been lacking, we suggest others deploy this further considering other minority stressors and samples.

While it may therefore be that gender identity moderates other minority stress variables, another future direction is to develop a more specific minority stress theory for adolescence. For instance, a core component of the theory is that the chronicity of stressors itself contributes to mental health inequalities. However, as noted in the introduction, gender identity is to some extent emerging, becoming more salient, and fluid in adolescence, meaning that effects linked specifically to chronicity would not necessarily be expected in the same way for adolescents as adults. In any case, particularly given modelling advancements since the theory was conceived, there is scope to develop tighter hypotheses, particularly taking into account developmental considerations.

### 5.4. Implications for policy and practice

Much more research is needed in this area. Nevertheless, the current analysis provides robust evidence of TGD youth as a particularly vulnerable group in terms of both increased exposure to stressors and poorer wellbeing outcomes. This highlights the urgent need for efficacious intervention that provides appropriate forms of support to meet their needs. McDermott *et al*. [92] argue that effectively tailored services should de-pathologize distress, difficult thoughts and behaviours; address normative environments that marginalize adolescents; and that professionals must understand individual lives, connect with adolescents and facilitate their sense of agency. We also know that TGD young people experience inequalities in terms of their access to health services [93]. Improving the accessibility and quality of targeted interventions alone is therefore insufficient, and we argue universal intervention is required. This includes work to reduce institutionalized forms of prejudice and discrimination, including policies and institutions that pathologize gender diversity, fail to recognize the value of gender affirmative care and/or create delays in access to healthcare through increased bureaucracy (e.g. unnecessary assessments and evaluation periods) [94,95].

Thinking more specifically about the high levels of gender discrimination experienced by our TGD group, our findings perhaps prompt scrutiny of the relationships and sex education statutory guidance for schools [96], and evaluation of the extent to which it is being implemented effectively. While coverage of gender identity issues is mandated in said policy, it could be argued that details are lacking, which may leave schools uncertain about how to address this sensitive topic. In this void, recently published guidance [94,97] may prove useful, spanning medical and legal considerations, related non-statutory guidance, and what appropriate provisions could include, all of which are aimed toward the development of a school environment that is inclusive, safe and respectful of TGD youth.

### 5.5. Strengths and limitations

The current study drew on a large sample, a robust and gender-invariant measure of wellbeing, and rigorous analysis strategy. Our work also benefits from the registered report format, with the increased transparency this affords. Nonetheless, we acknowledge several limitations. First, there were a couple of eventualities we had not foreseen in our registered analysis. We had not considered that we might have attrition in two directions (that is to say, new participants at T2, due in part to the unique way the #BeeWell study is embedded in Greater Manchester). Nevertheless, clear guidance for this scenario is not available to our knowledge and is probably best handled by full information maximum likelihood (FIML), in line with our existing pre-registered analysis strategy. In addition, we had not considered how we would report the structural model if found to be invariant, given that we expected gender moderation effects and used a strict omnibus test likely to err on the side of over-rejection in combination with the more lenient invariance criterion. Consequently, when we came to this null finding for H4, we considered the power gains of reporting the full model as a single group to be justified. Both of these issues also affect our *a priori* power simulation (since we anticipated 20% attrition in a single direction at T2). We argue our approach of reporting and interpreting the single group represents the simplest, most robust approach to reporting and interpretation. For transparency, the multi-group model implicitly consistent with our registered analysis is reported in the electronic supplementary material.

Second, we only considered the moderating effect of gender at T1, though gender identity is known to fluctuate in this period. While this was appropriate to implement temporal precedence, future work might somehow also take into account the fluid nature of gender identity in adolescence. Third, though large and close to national averages in terms of composition, the current sample can only be considered representative of Greater Manchester, meaning more work is needed to replicate this work in other samples. Finally, as noted above, since this was a secondary analysis we were limited to available minority stressors, and future work should consider alternatives drawing on reliable (multi-item) indicators where possible.

## 6. Conclusion

Despite a clear need to robustly estimate wellbeing inequalities across gender groups, work in large general population samples was lacking. We addressed this, drawing on best-practice recommendations for analysing gender, and using an invariant outcome measure. Stark disadvantages, at both time points, were found comparing TGD and CM adolescents for wellbeing and notably also gender discrimination. Though we did not find evidence to support a moderating effect of gender identity on minority stressors predicting later wellbeing, we demonstrated how methods to analyse this could be robustly applied and highlighted several future directions.

## Data Availability

An anonymized version of the #BeeWell survey responses will be made publicly available in 2026. Due to ethical constraints, this cannot be brought forward since participants have been given the right to withdraw their data until this point necessitating the need to maintain a securely stored pseudonymized version until this point. In addition, the non-self report data such as sex and free school meal eligibility will never be shared publicly due to the agreement in place with the local authorities who provided it. Analysis code is provided in the electronic supplementary material [98]. Per our timeline for the agreed period in which parents/carers and/or young people can request the deletion of their data, we will produce an anonymized dataset by the end of 2026 and deposit in a suitable repository (e.g. Open Science Framework). Following in-principle acceptance, the approved stage 1 version of this manuscript was pre-registered on the Open Science Framework at https://osf.io/w7x8m. This pre-registration was performed prior to data analysis. Simulated data is provided in the electronic supplementary material.

## Footnotes

1 In the original stage-one submission (https://osf.io/w7x8m), our unregistered hypothesis 2 was worded in such a way that it could not be operationalized by our submitted method. In the final version, we have therefore edited the wording of this hypothesis to reflect what could actually be achieved by the approved methods.

2 In the original stage-one submission (https://osf.io/w7x8m), the frequencies in this table are different from those presented here in the final stage-two version. This is for several reasons: first, the dataset was updated since a school later revealed they had delivered the survey to the wrong year group resulting in 142 fewer Y8 participants. Second, in the initial code, missingness on all was considered for only the variables in the model (i.e. gender and sex were not considered though some adolescents had data for these but not those in the model). This resulted in 496 participants who had some data across all variables at both time points and could be included. Therefore, based on 496 − 142, the overall number of participants with T1 data resulted in a discrepancy of 354 (more in the final analysis) between the originally reported analysis (and cis variable frequencies) and final stage-two analysis. It was also particularly appropriate to consider these additional participants since many also had data at T2. Based on these issues and for transparency, the T1 analyses were updated compared with the original stage-one submission. Results were substantively unchanged.

3 Though this scale can be treated as latent in larger models, considered on its own, the model is saturated since it has only three items and is therefore unsuitable for measurement invariance analysis.
